# *In vivo* efficacy proof of concept of a large-size bioprinted dermo-epidermal substitute for permanent wound coverage

**DOI:** 10.3389/fbioe.2023.1217655

**Published:** 2023-07-25

**Authors:** Maxime Abellan Lopez, Laurence Hutter, Etienne Pagin, Mélanie Vélier, Julie Véran, Laurent Giraudo, Chloe Dumoulin, Laurent Arnaud, Nicolas Macagno, Romain Appay, Laurent Daniel, Benjamin Guillet, Laure Balasse, Hugo Caso, Dominique Casanova, Baptiste Bertrand, Françoise Dignat, Loïc Hermant, Hélène Riesterer, Fabien Guillemot, Florence Sabatier, Jérémy Magalon

**Affiliations:** ^1^ Plastic Surgery Department, Hôpital de la Conception, AP-HM, Marseille, France; ^2^ Aix-Marseille Université, INSERM, Institut National de Recherche Pour l'Agriculture, l'Alimentation et l'Environnement, Centre de Recherche en Cardiovasculaire et Nutrition (C2VN), Marseille, France; ^3^ Poietis, Pessac, France; ^4^ Cell Therapy Department, Hôpital de la Conception, AP-HM, INSERM CIC BT 1409, Marseille, France; ^5^ Vascular Biology Department, Hôpital de la Timone, AP-HM, Marseille, France; ^6^ Anatomy and Pathology Department, INSERM U1263, C2VN, Hôpital de la Timone, Marseille, France; ^7^ Centre Européen de Recherche en Imagerie Médicale (CERIMED), Aix-Marseille Université, Centre National de la Recherche Scientifique, Marseille, France

**Keywords:** skin substitute, wound healing, multimodal bioprinting, laser-assisted bioprinting, large size, good manufacturing practice compatible

## Abstract

**Introduction:** An autologous split-thickness skin graft (STSG) is a standard treatment for coverage of full-thickness skin defects. However, this technique has two major drawbacks: the use of general anesthesia for skin harvesting and scar sequelae on the donor site. In order to reduce morbidity associated with STSG harvesting, researchers have developed autologous dermo-epidermal substitutes (DESs) using cell culture, tissue engineering, and, more recently, bioprinting approaches. This study assessed the manufacturing reliability and *in vivo* efficacy of a large-size good manufacturing practice (GMP)-compatible bio-printed human DES, named Poieskin^®^, for acute wound healing treatment.

**Methods:** Two batches (40 cm^2^ each) of Poieskin^®^ were produced, and their reliability and homogeneity were assessed using histological scoring. Immunosuppressed mice received either samples of Poieskin^®^ (*n* = 8) or human STSG (*n* = 8) immediately after longitudinal acute full-thickness excision of size 1 × 1.5 cm, applied on the skeletal muscle plane. The engraftment rate was assessed through standardized photographs on day 16 of the follow-up. Moreover, wound contraction, superficial vascularization, and local inflammation were evaluated via standardized photographs, laser Doppler imaging, and PET imaging, respectively. Histological analysis was finally performed after euthanasia.

**Results:** Histological scoring reached 75% ± 8% and 73% ± 12%, respectively, displaying a robust and homogeneous construct. Engraftment was comparable for both groups: 91.8% (SD = 0.1152) for the Poieskin^®^ group *versus* 100% (SD = 0) for the human STSG group. We did not record differences in either graft perfusion, PET imaging, or histological scoring on day 16.

**Conclusion:** Poieskin^®^ presents consistent bioengineering manufacturing characteristics to treat full-thickness cutaneous defects as an alternative to STSG in clinical applications. Manufacturing of Poieskin^®^ is reliable and homogeneous, leading to a clinically satisfying rate of graft take compared to the reference human STSG in a mouse model. These results encourage the use of Poieskin^®^ in phase I clinical trials as its manufacturing procedure is compatible with pharmaceutical guidelines.

## 1 Introduction

Full-thickness skin defects are mainly represented by acute carcinologic excisions, donor-site scarring of reconstructive procedures, burn injuries, and traumatic and chronic wounds. Patients suffering from these types of lesions need to be properly and promptly treated. An autologous split-thickness skin graft (STSG) is the reference treatment for skin coverage, especially when the defect is large and reaches the hypodermis. STSG of 200–500 µm thick is harvested using a dermatome and can be mechanically expanded for higher coverage capacity. In the case of severe burn injuries, it remains the only surgical solution to ensure patient survival, and surgeons need to manage with a donor-site shortage. However, this technique has some significant drawbacks such as the use of general anesthesia, leaving a noticeable scar on the donor site, and long-term stiffness (Voineskos et al., 2009).

Technological advances, including cell-based therapy and tissue engineering, have fostered the development of skin substitutes for temporary (Salisburry et al., 1973; Tavis et al., 1979; Ramakrishnan and Jayaraman, 1997; Pegg, 2002) and permanent wound coverage to overcome STSG limitations. *In vitro* manufacturing of the epidermal layer was initiated in 1975, when Rheinwald and Green managed to culture autologous keratinocytes from a small cutaneous biopsy (Rheinwald and Green, 1975). Since then, a large area of the epidermal layer can be produced by cell culture into keratinocytes sheets (Renner et al., 2009; Sheridan, 2009; Ortega-Zilic et al., 2010; Takami et al., 2014) or directly in a suspension to spray (Moustafa et al., 2007; Límová, 2010; Kirsner et al., 2012) on a vascularized ground. More challenging projects have proposed autologous dermo-epidermal skin reconstruction. Self-assembly manufacturing techniques have already been validated to produce large-size substitutes (from 35 to 45 cm^2^) used in burn injuries and chronic wound applications (NCT02145130, 2014; NCT03229564, 2017; NCT03394612, 2018; Boa et al., 2013; CHU de Quebec-Universite Laval, 2014; Germain et al., 2018; Auger, 2022; Blok et al., 2013; Blok, 2016; NCT01655407, 2016). However, this handmade manufacturing method remains difficult to implement in therapeutic and industrial applications in an automated manner.

In this context, 3D bioprinting offers clear advantages over conventional skin tissue engineering. This technology enables the generation of different functional tissues with appropriate architectures and cell compositions of different sizes with high throughput and reproducibility (Lee et al., 2009; Lee et al., 2010; Lee et al., 2010; Zhao et al., 2012; Baltazar et al., 2020) as it can print layer-by-layer different types of cells combined in a selected matrix. Because of its multilayered structure, skin is a perfect example of the strengths and benefits of 3D bioprinting approaches, overcoming some of the limitations of traditional tissue engineering strategies (Arslan-Yildiz et al., 2016; Miri et al., 2019; Yu et al., 2020). This approach has been investigated on animal models since 2013 for skin engineering (Cubo et al., 2016; Albanna et al., 2019; Baltazar et al., 2020; Jorgensen et al., 2020). Although these works formally use 3D bioprinting techniques, the manufacturing methods described (Lee et al., 2009; Cubo et al., 2016) are far from the good manufacturing practice (GMP) requirements necessary to bring this innovative technology to the clinical level. Furthermore, none of these studies manufactured a large-size dermo-epidermal substitute (DES) compliant with a potential therapeutic application in a GMP-compatible protocol compared with the current reference treatment, autologous STSG.

We developed a manufacturing process for a DES, named Poieskin^®^, based on the combination of extrusion bioprinting for biomaterials and laser-assisted bioprinting (LAB) for cell seeding, leveraging the advantages of high volumes and speed deposition of the matrix and high resolution and viability maintenance of cells. These two approaches were combined in the same equipment referred to as the Next-Generation Bioprinting (NGB) system designed to meet the regulatory GMP requirements for advanced therapy medicinal product (ATMP) manufacturing (EMA, 2018).

Herein, we report the reliability and homogeneity of a large-size (40 cm^2^) GMP-compatible DES, named Poieskin^®^, as well as its efficacy and safety in the treatment of acute wound healing on an immunosuppressed murine model as part of the Poieskin^®^ preclinical development program.

## 2 Methods

### 2.1 Ethics declaration

In this preclinical study, the authors manufactured batches of Poieskin^®^ under GMP-compatible conditions using cells harvested from patient biopsies. Human skin biopsies for Poieskin^®^ production and human split-thickness skin graft (HSTSG) controls were surgical residues removed during plastic surgeries on healthy volunteer patients (BIOPSKIN study number NCT04925323).

All animal experiments were conducted in accordance with the Aix-Marseille University Institutional Animal Care and Use Committee (CE14, Aix-Marseille University) and the French Ministry of Research (project authorization #33645 on 29 November 2021), according to the European Union directive 2010/63/EU and the recommendations of the Declaration of Helsinki. The study was approved by the National Animal Care and Ethics Committee (no. APAFIS 2020012015402650). Trained and authorized operators performed all animal experiments.

### 2.2 Human split-thickness skin

Poieskin^®^ wound healing efficacy was compared to HSTSG as a control group. Tissue was harvested from healthy donor patients during mammaplasty procedures (after obtaining written informed consent) using an electrical dermatome (Acculan 3Ti^®^ Dermatome, Aesculap, Hazelwood, MO, United States) set to harvest 0.2–0.3-mm-thick skin, according to the STSG defined thickness range. HSTSG was collected 1 hour before the grafting procedure and was transferred in a sterile container with 0.9% NaCl solution to the center for *in vivo* transplantation.

### 2.3 Poieskin^®^ biofabrication

Human dermal fibroblasts and epidermal keratinocytes were extracted from a skin biopsy. We used two 4-cm^2^ skin biopsies to produce two 40-cm^2^ skin substitutes. Keratinocytes and fibroblasts were isolated from human donor skin. The hypodermis compartment was manually deleted, and the skin biopsy was cut into small pieces. The epidermis and dermis were separated after overnight incubation at 4°C in 2.4 U/mL of dispase. Collagenase NB5 was used at a concentration of 0.25 U/mL for fibroblast isolation and trypsine 0.05% for keratinocyte separation.

The viability and concentration of keratinocytes and fibroblasts in viable nucleated cells were checked after each trypsination using an automated cell counter Luna X7 (Logos Biosystems, Villeneuve-d’Ascq, France).

After primary seeding, keratinocytes and fibroblasts were detached using trypsin‐EDTA and cryopreserved in 10% DMSO + 90% FBS for storage at −150°C. For tissue production, keratinocytes and fibroblasts were thawed and further cultured and amplified *ex vivo*.

Keratinocytes were grown on an allogeneic feeder layer of irradiated human fibroblasts (WCB-3C, AP-HM, Marseille, France) and cultured in a keratinocyte medium containing 58% Dulbecco–Vogt modified Eagle’s medium, 30% Ham’s F12, 2 mM GlutaMAX, 10% bovine FetalClone II serum, 10 ng/mL human epidermal growth factor, and 20 μg/mL gentamicin.

Fibroblasts were grown in fibroblast media containing alphaMEM, 5% platelet lysate, 2 UI/mL heparin, 100 U/mL penicillin, and 20 μg/mL gentamicin.

In preparation for dermis bioprinting, human dermal fibroblasts (passage 3) were suspended in DPBS to obtain a bioink containing 12.5 million viable nucleated cells/mL for LAB. Meanwhile, bovine collagen ink was formulated at 4 mg/mL using 10X DPBS, water for injection (WFI), and NaOH for extrusion bioprinting at 4°C. Dermal architecture consisted of depositing three layers of collagen and fibroblasts by extrusion and LAB, respectively.

After bioprinting, the dermis was cultured for 5 days in dermis maturation media containing alphaMEM supplemented with 3% platelet lysate, 2 UI/mL heparin, 100 U/mL penicillin, 20 μg/mL gentamicin, and 50 μg/mL laroscorbine.

Keratinocytes (passage 2) were suspended in DMEM to obtain a bioink containing 70 million viable nucleated cells/mL for LAB.

After bioprinting keratinocytes onto the bioprinted dermis, the skin constructs were cultured under immersed conditions for 2 days in a dedicated medium containing 58% Dulbecco–Vogt modified Eagle’s medium, 30% Ham’s F12 , 3% platelet lysate, 10 ng/mL human epidermal growth factor, 100 U/mL penicillin, 20 μg/mL gentamicin, and 50 μg/mL laroscorbine.

**FIGURE 1:**
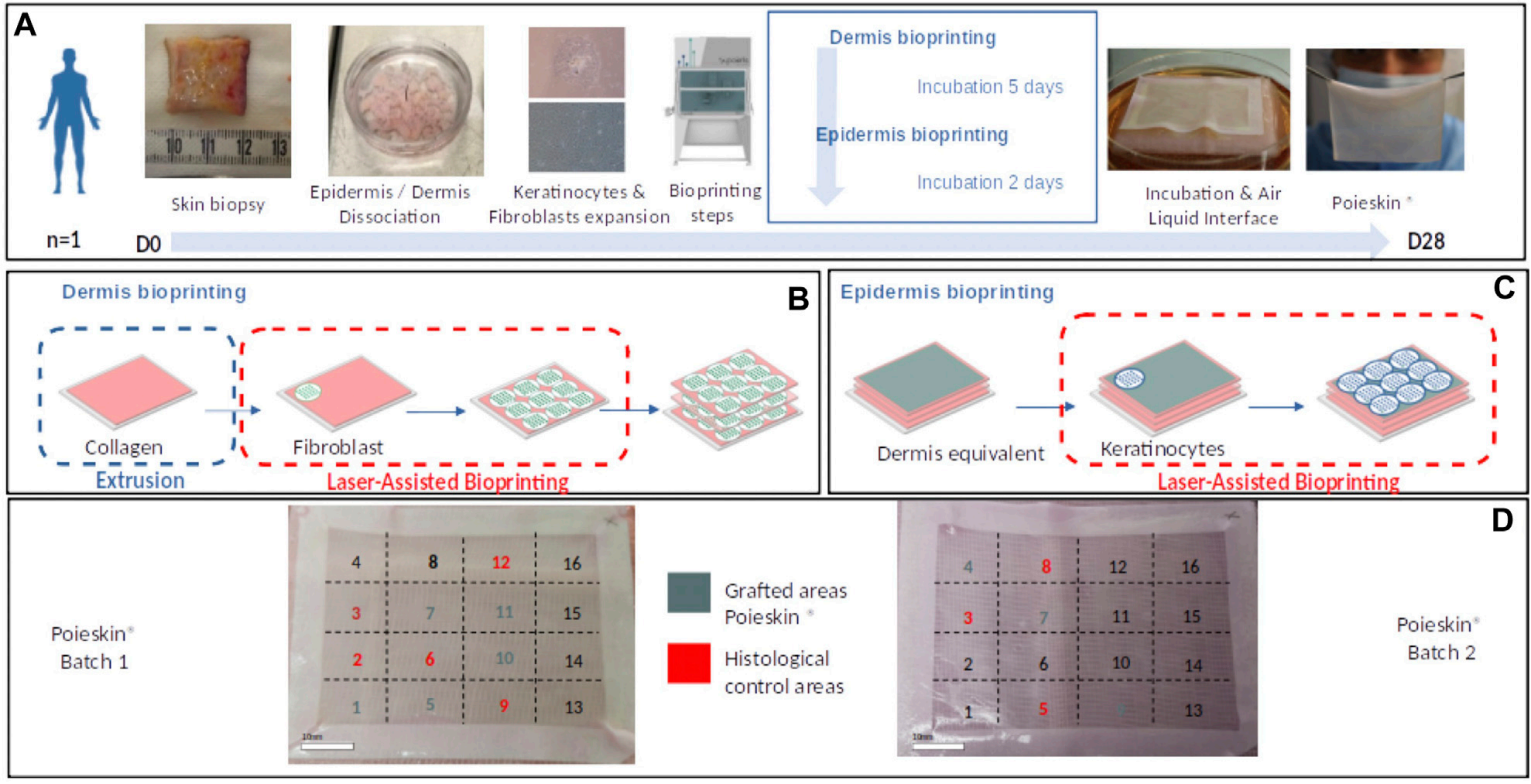
**(A)** Summary of main Poieskin^®^ manufacturing steps. **(B)** Dermis bioprinting steps: successive bioprinting of collagen layers with extrusion bioprinting and fibroblast layers with laser-assisted bioprinting (LAB). **(C)** Epidermis bioprinting steps: keratinocyte deposition with LAB. **(D)** Final repartition of Poieskin^®^ product batches 1 and 2 between histological control and grafts on mice.

Cells were expanded before being formulated into bio-inks and bioprinted using an NGB bioprinter, in a GMP-compatible process, which has been defined by the use of GMP reagents and culture media for all the steps of the Poieskin^®^ manufacturing process and the conformity to the acceptance criteria on the final Poieskin^®^ product (size = 40 cm^2^ ± 10%; dermal thickness >200 μm; histological score >50; and sterility of culture media including the specific absence of mycoplasms). Two 40-cm^2^ DESs were produced through a multistep protocol (Figure 1A). First, the multilayer dermal compartment was sequentially obtained by printing human primary fibroblast patterns on a bovine collagen (SYMATESE, Chaponost, France) layer printed by bioextrusion (Figure 1B). Second, the epidermal compartment was obtained by printing human primary keratinocyte patterns on the dermal equivalent (Figure 1C). Then, both 40-cm^2^ DESs were matured at the air–liquid interface (ALI) for the final epidermal differentiation for 6 days with a culture medium containing 58% Dulbecco–Vogt modified Eagle’s medium, 30% Ham’s F12, 3% platelet lysate supplemented with 0.8% human serum albumin, 0.4 μg/mL hydrocortisone, 0.12 UI/mL insulin, 100 U/mL penicillin, and 20 μg/mL gentamicin. We prepared a 1.6X ALI medium and then mixed it with agarose to obtain a final medium of 1X ALI medium and agarose 0.75%. We allowed it to gelify and then placed the Poieskin on top of it (it does not sink in the gelified medium) so that the air–liquid interface is maintained.

On day 6 of the ALI, the DESs were placed on gelified ALI media at room temperature for 6 h and transported from the manufacturing facilities (Poietis, Pessac, France) to the animal facilities (CERIMED, Marseille, France) for grafting. Before grafting, the DESs were cultured at the ALI with fresh ALI media for 24 h. Throughout fabrication, in-process characterizations were performed. Eight areas of 1.5 cm^2^ were selected for the grafting procedure, and eight additional areas of the same dimensions were used for control tests (Figure 1D).

Mechanical behavior was assessed by a senior plastic surgeon whose conformity was defined to be robust enough not to break when packaged, manipulated, and applied to the surgical site.

### 2.4 Homogeneity and quality control of Poieskin^®^ biofabrication

Control samples from 40-cm^2^ Poieskin^®^ to be grafted were analyzed to characterize the production features, as well as sections of the wound samples 16 days after grafting. Thickness measurements were made on the histology images using ImageJ software (National Institute of Health, Bethesda, United States).

The quality of the DES was assessed from histological sections after staining with Masson’s trichrome (described in Section 2.11). *POIETIS* developed a scoring grid to evaluate dermal and epidermal structures, excluding skin appendages, in order to quantitatively assess the quality of the evaluated samples. The semiquantitative score and associated parameters are presented in Supplementary Table S1.

### 2.5 *In vivo* preclinical study design

**FIGURE 2:**
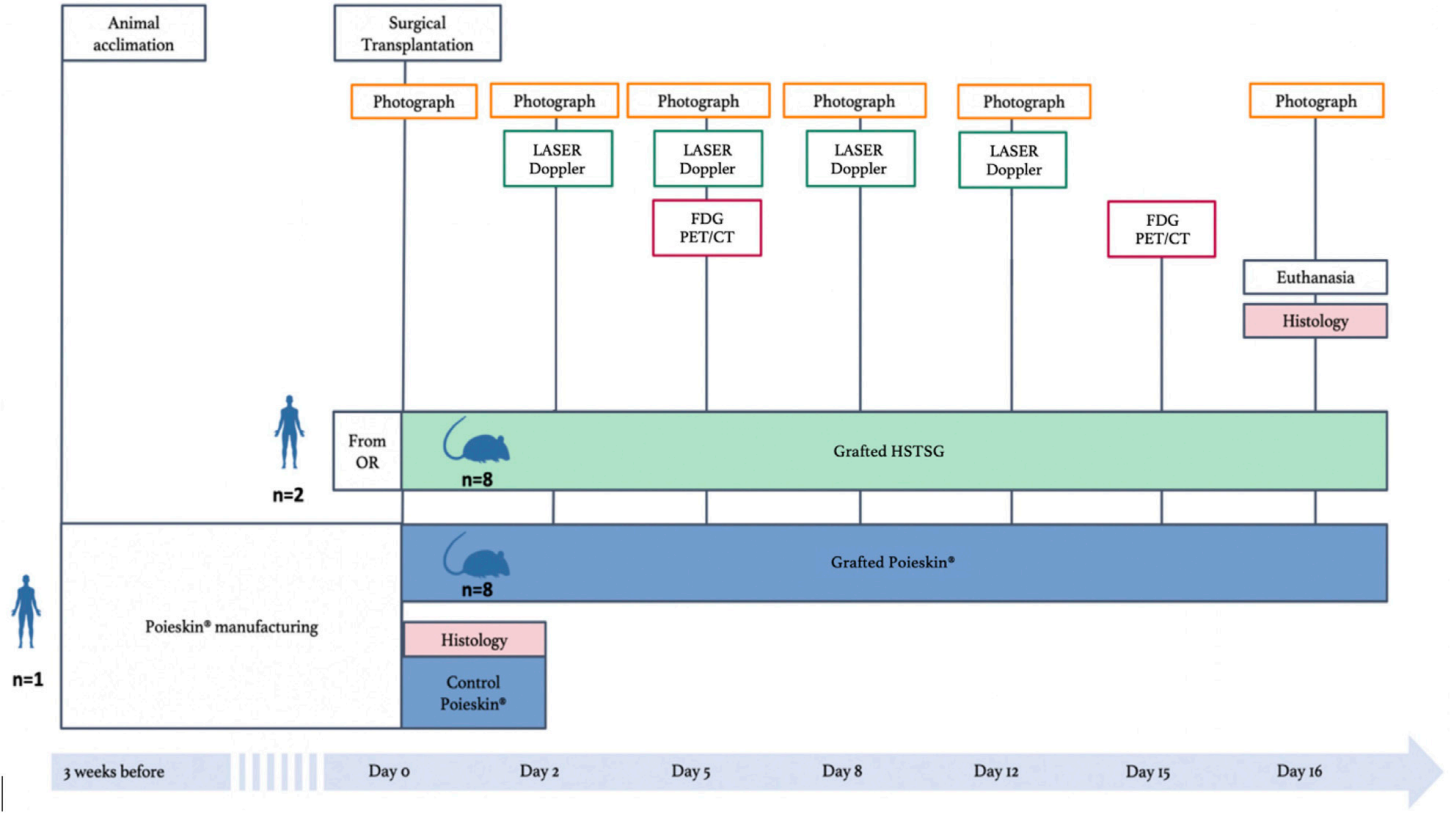
Study design comparing human split-thickness skin graft (HSTSG) to Poieskin^®^ (OR, operating room).

The study was designed to demonstrate the non-inferiority of Poieskin^®^ compared to the reference HSTSG in a relevant animal model (Figure 2). Immunosuppressed female NMRI-Foxn1^nu/nu^ mice (aged 7 weeks and weighing 20–23 g) were purchased from Janvier Laboratories (Genest-Saint-Isle, France). They were allowed to acclimatize for 2 weeks before the experiments. Eight mice received a Poieskin^®^ graft, while eight others received HSTSG on their back immediately after a cutaneous excision of 1 × 1.5 cm. The primary endpoint was the area measurement of the graft taken on standardized photographs in a 16-day follow-up. Moreover, the following secondary endpoints were evaluated: area percentage of wound contraction, superficial vascularization assessed by laser Doppler imaging, local inflammation by PET imaging, and histological analysis after euthanasia.

### 2.6 Surgical transplantation

The initiation of the surgical procedure in mice was synchronized to the end of Poieskin^®^ maturation and surgical harvesting of HSTSG, in order to perform both treatments on the same day, without delay, under comparable conditions. The mice received a subcutaneous injection of buprenorphine at a dose of 0.1 mg/kg 30 min before general anesthesia by sevoflurane initiation in an induction chamber (8%; 1 L/min). Anesthesia was maintained via a nose cone (2.5%–3%; 0.5 L/min) with spontaneous ventilation on an electric heater carpet at 37°C. Protective collyrium was then administered. The procedure began by cleaning with iodine after marking a 1 × 1.5 cm longitudinal rectangular cutaneous area to excise on the back of the mice. Before incision, the mice received long-term local analgesia by a subcutaneous back-forward injection of ropivacaine at 4 mg/kg around the surgical site. Then, surgical ×2.5 magnifying loupes were used in the surgical procedures.

The surgical protocol consisted of three steps: i) realization of an acute full-thickness longitudinal 1 × 1.5 cm excision using a scalpel no. 15. Any continuous bleeding was coagulated using bipolar forceps. Scarring was followed by immediate covering (with Poieskin^®^ or HSTSG). Grafts were custom-sized according to the defect (1.5 cm^2^) from larger samples, using a scalpel no. 23, withdrawn from a 40-cm^2^ Poieskin^®^ specimen, or from a fresh human split-thickness skin sample for the HSTSG group. They were manipulated using microsurgical tweezers (without teeth); ii) application of an immediate graft on the skeletal muscles by plan and fixation surgical methods to the skin with 14 separate Prolene^®^ 6–0 (Ethicon Inc., Johnson & Johnson, New Brunswick, NJ, United States) stitches using a microsurgical holder and scissors; iii) application of custom-sized dressing on the graft of a non-adherent, non-absorbent UrgoTul^®^ interface (Laboratoires URGO, Chenôve, France) and then a non-woven surgical pad was fixed using an adhesive Tegaderm^®^ bandage (3 M, Saint Paul, MN, United States). We checked before awakening that the dressing did not induce any hindrance to chest expansion, limb mobility, and fecal or urinary tract.

During postoperative surveillance, the mice were placed under a heating lamp until awakening and then placed into an individually ventilated cage in a loose housing room. The general health condition was monitored daily. Analgesic subcutaneous injections of buprenorphine (0.1 mg/kg/day for the first week and then 0.05 mg/kg/day) could be administered, if necessary. No antibiotic was administered. Dressings were first changed on day 2 and then on days 5, 8, 10, 12, and 15 under general anesthesia.

### 2.7 Photograph analysis

Standardized photographs were taken preoperatively (day 0) on days 2, 5, 8, and 12 and finally on day 16 immediately before the mice were euthanized. The camera (Canon PowerShot SX220 HS, Tokyo, Japan) was placed vertically 35 cm above the operating site, and the view was zoomed to ×2, taking a centimeter ruler in the range in order to harmonize the area measurement performed using ImageJ software (National Institute of Health, Bethesda, United States). We analyzed the evolution of engraftment and retraction ratios. The engraftment ratio is defined as the vascularized epithelialized graft area divided by the total scarring area at the same time. On the other hand, the retraction ratio is calculated as the total scarring area at a specific moment divided by the total scarring area on day 0.

### 2.8 Graft perfusion analysis

Laser Doppler perfusion imaging (PIM2, Perimed, Craponne, France) was used to assess skin graft perfusion after surgery on days 2, 6, 12, and 16 post-surgery under sevoflurane anesthesia. The mice were induced with 8% sevoflurane in air, followed by 3% sevoflurane in air, and placed on a 37°C heated carpet. The results were expressed as an optical density ratio of the graft area to a healthy back skin area.

### 2.9 [18F]-FDG MicroPET/CT imaging

The mice were injected with 6.4 ± 1.5 MBq/100 µL [18 F]-FDG in the caudal vein on days 5 and 15 after surgery. MicroPET/CT images were acquired 60 min after injection during a 20-min imaging session. MicroPET/CT imaging was performed using a nanoScan PET/CT camera (Mediso, Budapest, Hungary) under isoflurane anesthesia. The mice were induced with 4% isoflurane in air, followed by 1.5% isoflurane in air (Isovet from Piramal, Voorschoten, Netherlands). Quantitative region-of-interest (ROI) analysis of the PET signal was performed on attenuation- and decay-corrected PET images using VivoQuant software v4.0patch1 (Invicro, Boston, MA, United States). Tissue standardized uptake values were expressed as the ratio of the graft area to the healthy back skin area.

For both techniques (laser Doppler and [18F]-FDG MicroPET/CT), the screened areas were divided into several ROIs. Unaffected skin on the neck and lumbar area served as controls to assess vascularization and local inflammation evolution of the grafts.

### 2.10 Histological analysis and immunostaining

On day 0, control DESs were fixed with 4% (w/v) formaldehyde for 8 h at room temperature. At the end of the 16-day follow-up, the mice were euthanized with a lethal overdose of sevoflurane inhaled into an induction chamber associated with cervical dislocation. The surgical sites were then entirely harvested, taking laterally 3-mm-width margins and vertically from the underlying musculoskeletal system to the cutaneous envelope, in order to collect an exhaustive sample for histological analysis. Sections of the wound samples were immediately fixed with 4% (w/v) formaldehyde at room temperature for 48 h and maintained at 4°C for 72 h.

### 2.11 Histology and immunostaining

Fixed samples were dehydrated in successive ethanol and xylene solutions prior to paraffin embedding using a Histokinette (Leica, Wetzlar, Germany). Sections of 5 μm were generated using a microtome (Leica) and then stained with Masson’s trichrome dyes before imaging by light microscopy (Eclipse Ts2, Nikon, Tokyo, Japan). The histological characteristics of all grafts, Poieskin^®^ and HSTSG, were analyzed by an independent skilled pathologist who was blinded to the samples, using NDP2 viewing software (Hamamatsu Photonics, Hamamatsu, Japan) and a semi-quantitative scoring system as previously described.

Previously processed slides were permeabilized with 1X citrate buffer at pH 6 for 20 min at 98°C. Non-specific interactions were limited by incubation with a 2% (w/v) BSA-DPBS solution (bovine serum albumin, Merck, Burlington, Massachusetts, United States; Dulbecco’s phosphate-buffered saline, Dutscher) for 10 min. The samples were then incubated overnight at 4°C with primary antibodies, namely, K5 (1/200, Abcam, AB52635), K10 (1/200, Merck Millipore MAB3230), Coll1 (1/500, Abcam, Ab138492) (specific for human and bovine collagen 1 and does not stain mouse collagen 1), αSMA (1/200, Abcam, Ab5694) (Prost-Squarcioni et al., 2008) (Ruiter et al., 1993), and loricrin (1/200, Abcam, Ab85679) antibodies (Matsui and Amagai, 2015). After washing, secondary goat anti-rabbit Alexa Fluor 488 (1/200, Thermo Fisher Scientific, 10729174), secondary goat anti-rabbit Alexa Fluor 594 (1/200, Molecular Probes, R10477), and goat anti-mouse Alexa Fluor 488 (1/200, Thermo Fisher Scientific, 10256302) antibodies were added for 1 h at room temperature. The samples were counterstained with DAPI (1/1000, PK-CA707-40043, PromoCell, Heidelberg, Germany). Immunofluorescence images were captured using the Eclipse Ts2 microscope (Nikon) and analyzed using ImageJ software (National Institute of Health, Bethesda, United States).

The protein structure of the samples was analyzed by immunohistochemistry. Bovine type I collagen, which was used as a biomaterial in Poieskin^®^, allowed for the identification of the location of Poieskin^®^ and HSTSG. Cytokeratin 5 staining highlighted basal- and spinous-layer keratinocytes of human origin. The association of K10, K5, and loricrin staining revealed the epidermal maturation gradient, as they represent the basal, supra-basal, and corneal layers, respectively. αSMA was used to identify myofibroblasts or pericytes that could reveal neovascularization with a circular shape surrounding a lumen.

### 2.12 Statistical analysis

Statistical analysis was performed using GraphPad Prism 4.0 (GraphPad Software, La Jolla, CA, United States). The data that support the findings of this study are available on request from the corresponding author. The normality of data distributions was assessed using Shapiro–Wilk and Kolmogorov–Smirnov tests, and all data passed the normality test. Two-way ANOVA statistical tests were used for the analysis with multiple factor interactions (FDG-CT, graft take, and graft retraction). The engraftment ratio was defined as the vascularized epithelialized graft area divided by the total scarring area at the same time, whereas the retraction ratio corresponded to the total scarring area during evaluation divided by the total scarring area on day 0. A mixed-effect analysis was used for the analysis of laser Doppler imaging because values for performing two-way ANOVA were missing. Ultimately, ordinary one-way ANOVA was used for comparing the histological scorings. Graphs display the mean ± standard deviation (SD).

## 3 Results

**FIGURE 3:**
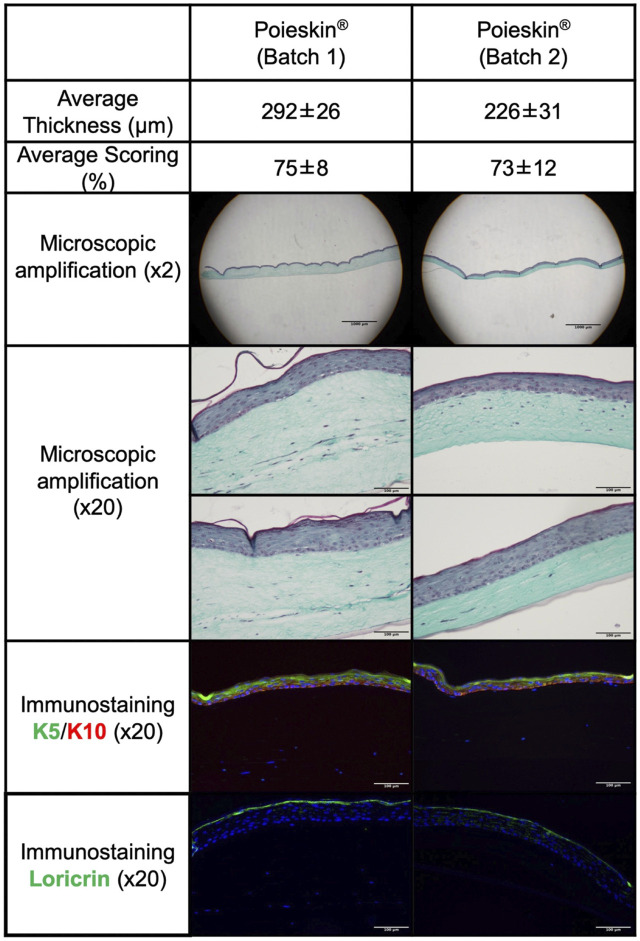
Histological and immunostaining characterization of Poieskin^®^ manufacturing: Poieskin^®^ batch 1: area 6 control of mouse 2, scoring = 78, and thickness = 355 ± 33 μm; Poieskin^®^ batch 2: area 3 control of mouse 6, scoring = 81, and thickness = 219 ± 45 µm.

The cells were harvested from a skin biopsy of a 25-year-old woman without co-morbidities (BMI was 24.6 kg/m^2^). The obtained Poieskin^®^ batches were intact after shipment and easy to manipulate after the final differentiation. All defined acceptance criteria were compliant for both final Poieskin^®^ batches. The total thickness of Poieskin^®^ grafts in two batches was 292 ± 26 μm and 226 ± 31 µm, respectively, which were considered optimal for grafting compared with HSTSG. According to the assessed scoring parameters of the dermal, epidermal, and basement membrane features, the histological structure of Poieskin^®^ was of reliable quality, with an average score of 75% ± 8% for batch 1 (*n* = 5) and 73% ± 12% for batch 2 (*n* = 3), well above the 50% compliance threshold. Samples within each batch were homogeneous regardless of their initial localization. Immunohistochemistry with anti-cytokeratin 5 and 10 revealed the presence of a proliferative layer of keratinocytes, as well as satisfactory differentiation of the upper layers of the epidermis, characterized by switching from cytokeratin 5 to 10. Furthermore, immunohistochemistry with anti-loricrin demonstrated the presence of a well-defined stratum corneum (Figure 3). Detailed information about the viability, doubling population, and concentration in viable nucleated cells before bioprinting and scoring and histological images are given in Supplementary Table S2 and Supplementary Figure S1, respectively.

**FIGURE 4:**
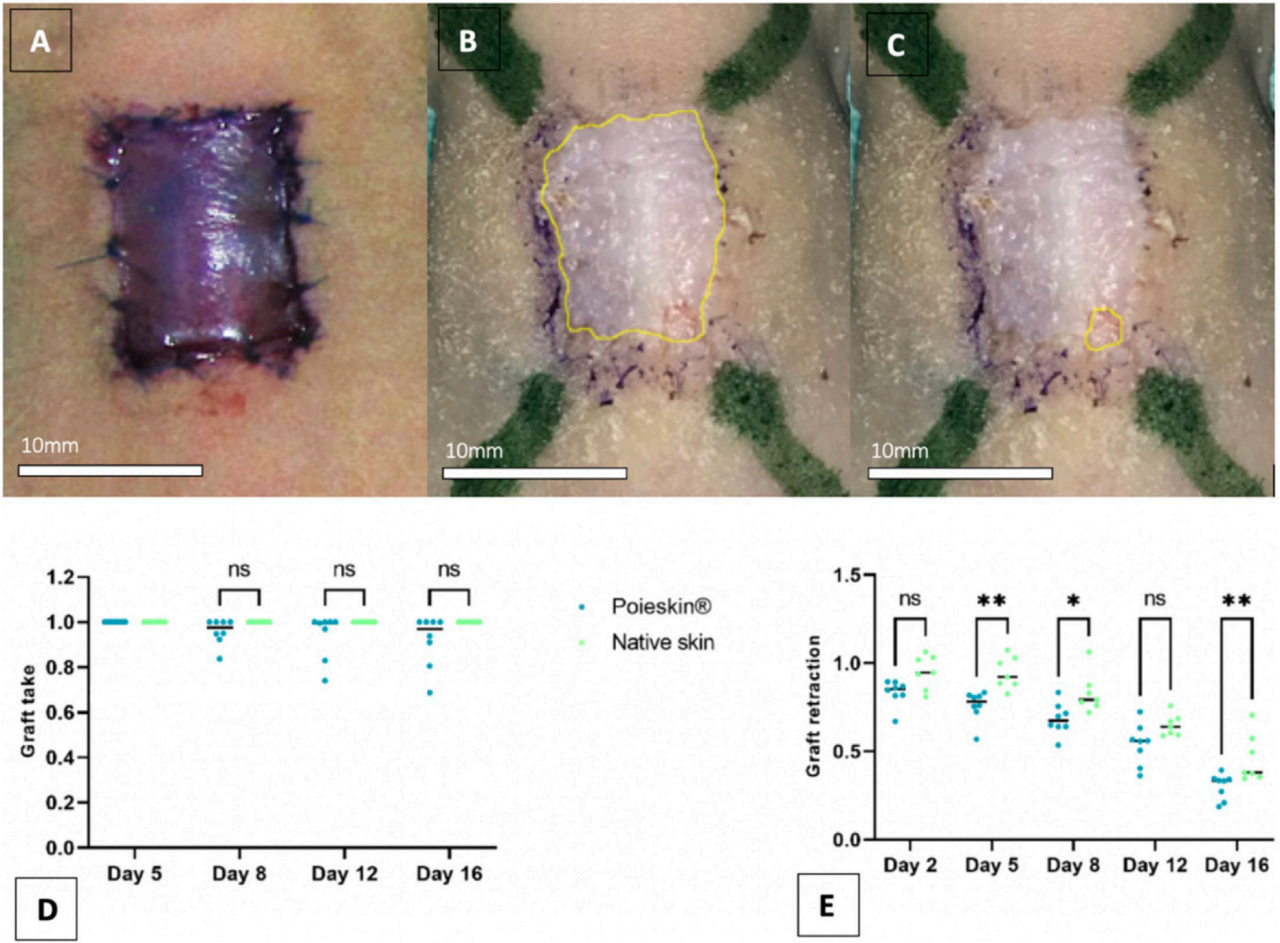
Grafted area measurements on standardized photographs (**(A)** Photograph of grafted Poieskin^®^ on day 0; **(B)** photograph of grafted Poieskin^®^ on day 16 with contouring of the total scarring area; **(C)** photograph of grafted Poieskin^®^ on day 16 with contouring of the graft lysed area; **(D)** assessment of graft take; **(E)** assessment of graft retraction; HSTSG, human split-thickness skin graft).

A total of 16 subjects (*n* = 16) were included receiving either HSTSG (*n* = 8) or Poieskin^®^ (*n* = 8). One mouse which received HSTSG died during surgery. HSTSG donor patients were 67- and 44-year-old women without co-morbidities (BMI was 27.0 kg/m^2^ and 27.3 kg/m^2^, respectively). The conformity with the mechanical behavior was observed for both batches, as shown in Supplementary Video S1 *.* Based on ImageJ software analysis, the mean graft take percentage was 91.8% (SD = 0.1152) on day 16 for Poieskin^®^ and 100% (SD = 0) for HSTSG (Figure 4). We did not observe a significant difference in graft take between the two groups (mean difference: −0.08225; 95% CI (−0.2177 to 0.05315); *p* = 0.2942). The retraction evolution of grafts ran faster for Poieskin^®^ than for HSTSG with a significant difference on day 16 (mean difference: −16.0%; 95% CI (−29.04 to −29.15); *p* = 0.0095). An example of the macroscopic aspect suggesting the quality of graft take, the difference in pigmentation, and the pliability is presented in Supplementary Video S2 **.**

**FIGURE 5:**
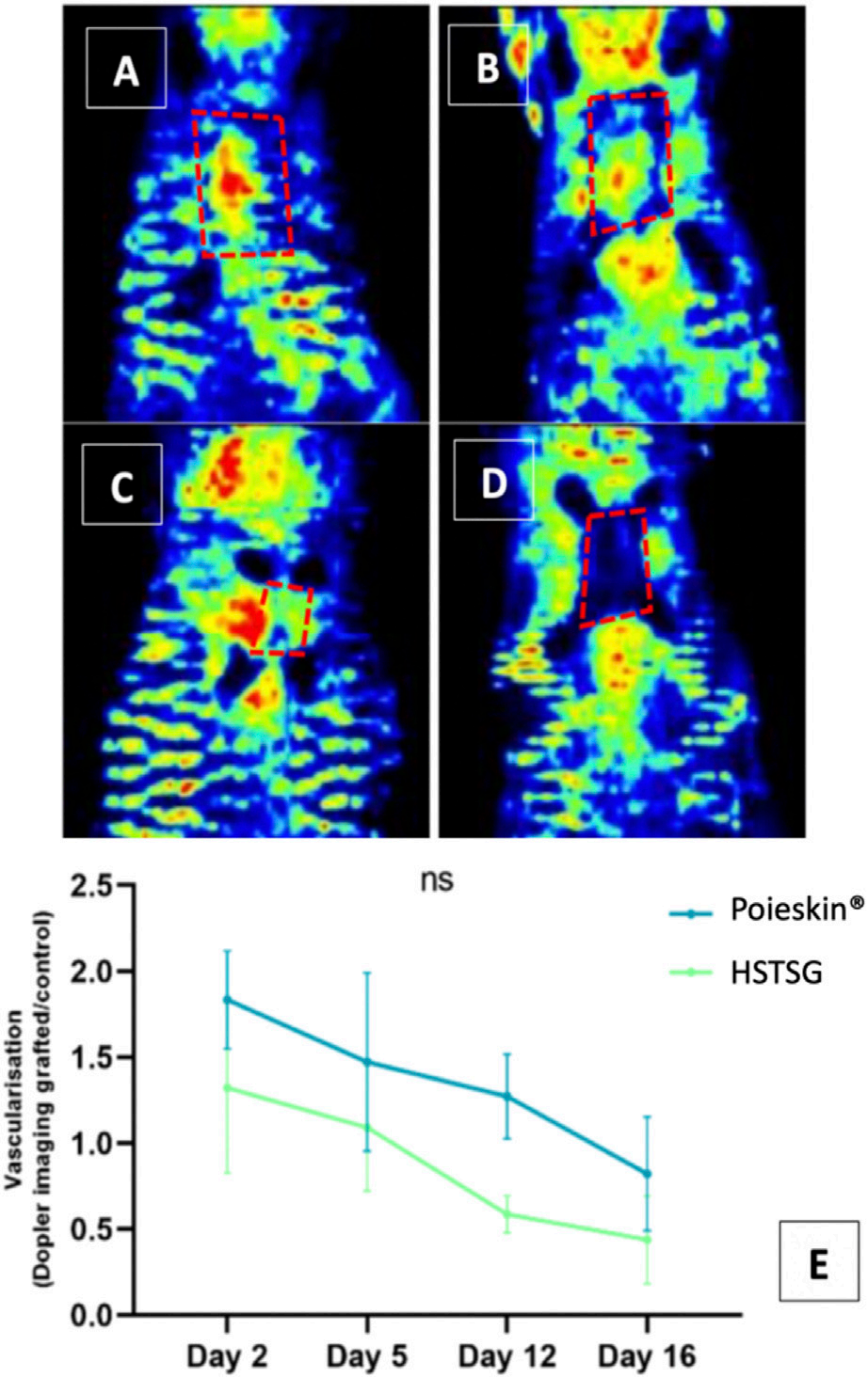
Graft perfusion assessment by laser Doppler (**(A)** Poieskin^®^ condition on day 2; **(B)** HSTSG condition on day 2; **(C)** Poieskin^®^ condition on day 16; **(D)** HSTSG condition on day 16; **(E)** evolution of the optical density ratio for both conditions).

**FIGURE 6:**
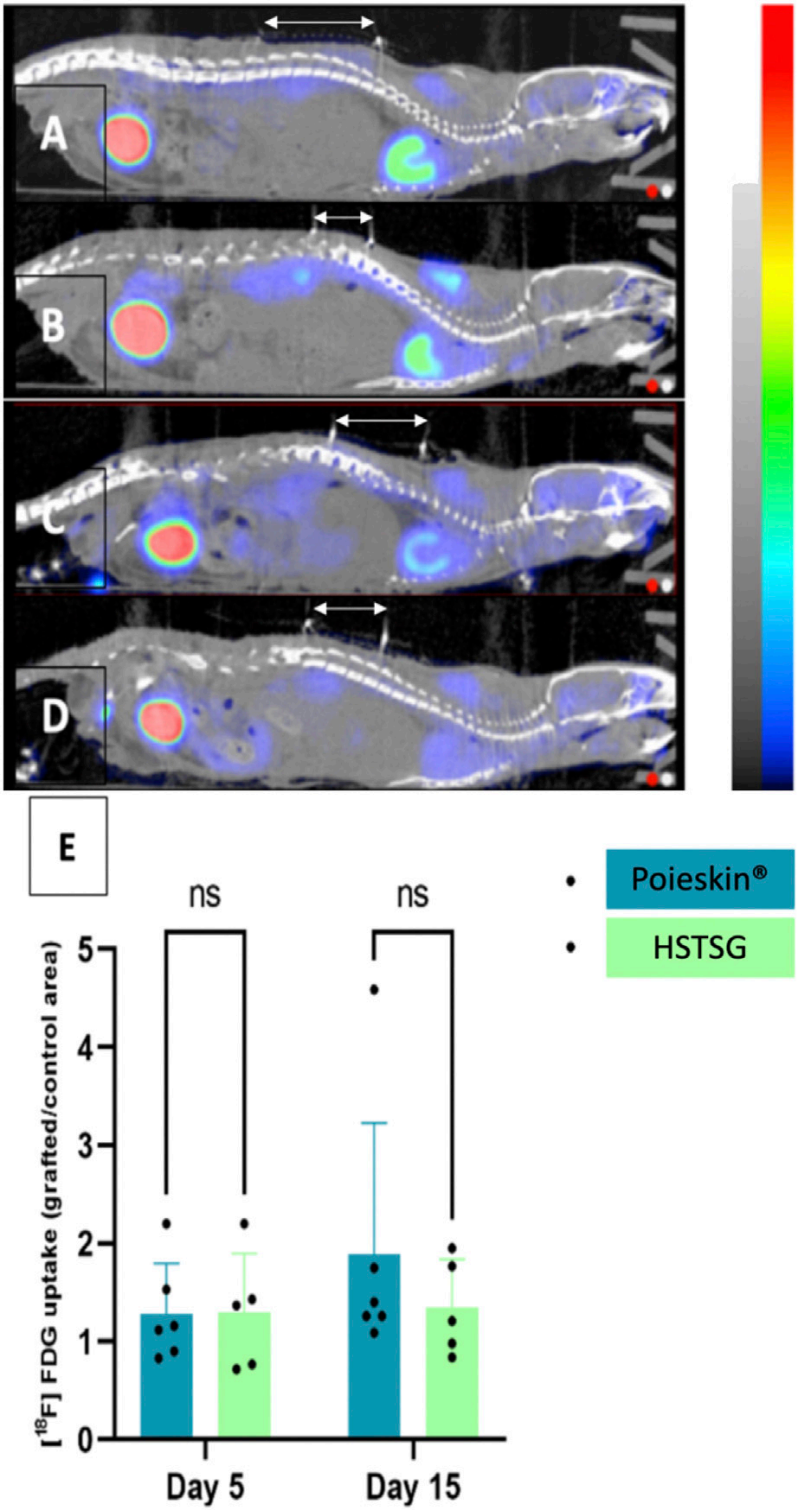
[18F]-FDG MicroPET/CT imaging (CT signal from −500 to 3,500 HU; PET signal from 0 to 8 Bq) 1 h after IV injection. Grafts are located below the white arrows (**(A)** Poieskin^®^ on day 5; **(B)** HSTSG on day 5; **(C)** Poieskin^®^ on day 15; **(D)** HSTSG on day 15; **(E)** metabolic activity measurements).

**FIGURE 7:**
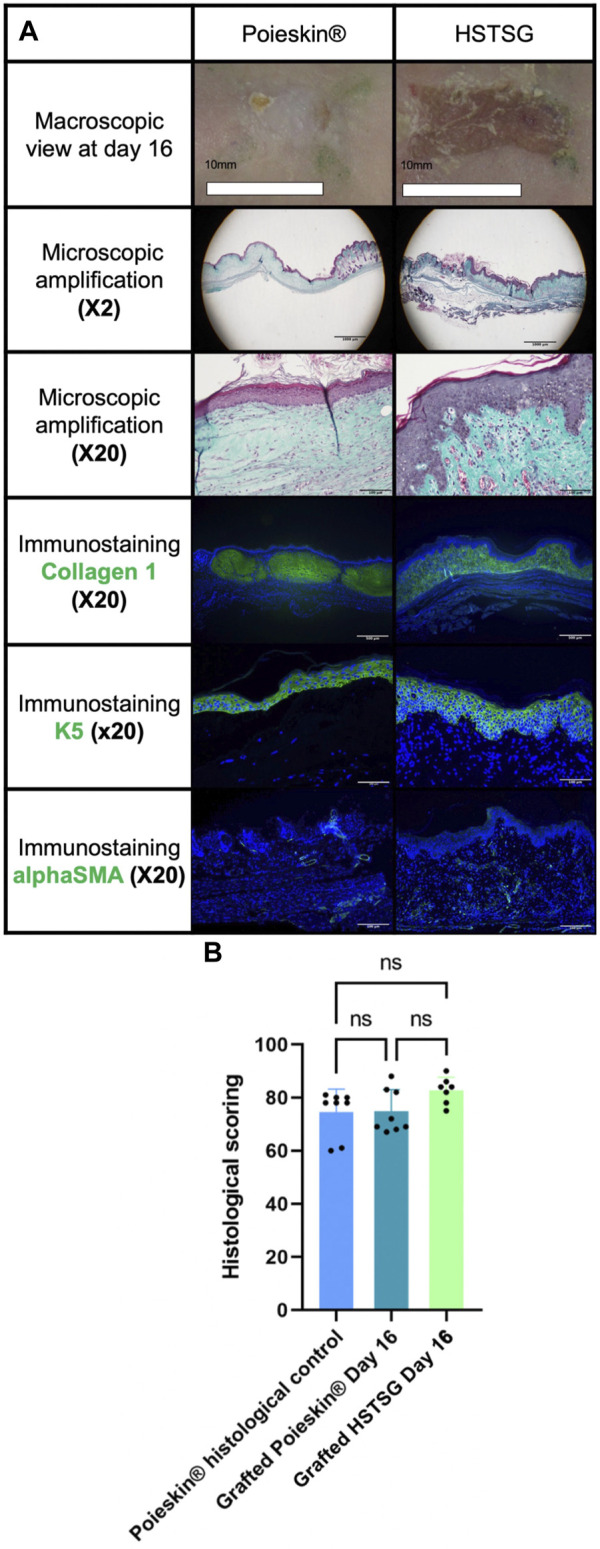
Results of histological analysis. **(A)** Representative images of the histological aspects of Poieskin^®^ (mouse 8, batch 2, area 9, scoring = 69, and thickness >300 µm) and human split-thickness skin graft (HSTSG) (mouse 9, scoring 84, and thickness >300 µm); **(B)** exhaustive results of histological scoring for Poieskin^®^ control, grafted Poieskin^®^, and grafted HSTSG.

Cutaneous perfusion assessment by laser Doppler imaging showed that the global optical density of the graft decreased over time (Figure 5). However, no significant difference was observed between the two groups (*p* = 0.1951 on day 16). No deaths, adverse events, clinical inflammation, or infection were observed during the entire procedure. Local inflammation assessment by [18F]-FDG MicroPET/CT imaging did not reveal any increasing metabolic activity in the grafted area (Figure 6). We did not observe any significant differences between Poieskin^®^ and HSTSG on either day 5 (*p* = 0.9998) or day 15 (*p* = 0.5109). Collagen 1 staining confirmed the presence of both Poieskin^®^ and HSTSG *in situ* 16 days after grafting on mice. On the other hand, mouse collagen was not stained as expected. Cytokeratin 5 staining highlighted basal and spinous layer keratinocytes of human origin. ɑSMA was used to identify myofibroblasts or pericytes that could reveal neovascularization with a circular shape surrounding a lumen. Based on our specific histological scoring, the mean score of Poieskin^®^ was 75% (SD = 8.31; *n* = 8), and the mean score of HSTSG was 83% (SD = 4.99; *n* = 7) (Figure 7). We did not record a significant difference in scoring between Poieskin^®^ and HSTSG with a mean difference of 7.964 (SD = 3.588) points (95% CI = 0.2121 to 15.72; *p* = 0.2423).

## 4 Discussion

This study demonstrated for the first time the reliability and homogeneity of a bioprinted large-sized DES under GMP-compatible conditions.

The NGB multimodal bioprinting manufacturing equipment successfully delivered functional DESs for *in vivo* grafting. The manufacturing process was completed within 28 days of initial biopsy procurement, and the substitutes’ thickness met the defined specifications set by clinicians. Histology and immunostaining analyses confirmed the presence of mandatory skin structures related to dermal and epidermal compartments.

Indeed, we were able to obtain two 40-cm^2^ batches with similar histological characteristics. The histological scoring was previously validated on more than 150 histological skin models (data not shown) by four independent operators to select and adjust the appropriate criteria and thresholds for the final version. In this study, the scoring grid was then used by an independent pathologist to calculate the score of the Poieskin batches.

We also assessed the safety and efficacy of Poieskin^®^ in a relevant immunosuppressed mouse model compared to the reference method, HSTSG.

Inflammation, as an expression of local infection, was monitored by TEP-CT FDG. We did not observe any increment and recorded variations that were close to the background signal. As no side effects occurred during the study, we could infer that Poieskin^®^ grafting is a safe procedure.

Furthermore, we reported an average engraftment of Poieskin^®^ of 91.8% 16 days after grafting, which was similar to the reference method. Qualitative observations of the grafted Poieskin^®^ samples showed that some microscopic aspects of HSTSG, which were not included in the printing pattern, appeared on day 16, such as intra-dermal micro-vessels, dermal papilla, and a coherent morphological continuum of the epidermal layer at the scar border. This endpoint is encouraging, and we assume that Poieskin^®^ could be an alternative to STSG in superficial full-thickness skin defects which are mainly represented by acute carcinologic excisions, donor-site scarring of reconstructive procedures, burn injuries (from deep second to third degree), and traumatic and chronic wounds. These indications are nowadays properly treated by 200–300 µm autologous STSG. The cutoff ratio deeming the success or failure of skin graft take is not consensual. It depends on intrinsic and extrinsic factors (such as the location, thickness, or type/etiology of the defect) and varies from 50% to 90% in the literature (Birchall et al., 1991; Kirsner et al., 1995; Thourani et al., 2003; Shores et al., 2012; Reddy et al., 2014; Lauerman et al., 2018; Braza and Fahrenkopf, 2022). In clinical applications of skin grafts, we consider an engraftment of over 80% of the graft area on days 15–21 as successful since the patient does not require additional procedures. In this study, we observed that seven out of eight mice grafted with Poieskin^®^ were above this 80% cutoff on day 16.

The skin graft area is known to shrink over time due to fibroblasts differentiating into myofibroblasts, inducing contraction. This is sometimes a desired effect as it decreases the size of the lesion, but it can stiffen adjacent articulations. Here, we recorded differences in the kinetics of area reduction: Poieskin^®^ showed faster contraction than HSTSG, reaching approximately 69% of contraction *versus* 54% for HSTSG, from day 5. According to our assumption, a poorer reticulation of the constructed dermis in Poieskin^®^ could induce faster graft dehydration *in situ* than that in HSTSG. The phenotype and organization of fibroblasts in the dermis could also be involved in this contraction phenomenon (Douillet et al., 2022).

Nevertheless, the Poieskin^®^ texture turned out to be more elastic but without pigmentation compared to HSTSG, with a softer appearance. Although not significant, this observation was consistent with the difference in the optical density on the laser Doppler assessment between Poieskin^®^ and HSTSG. Indeed, the lower optical density of HSTSG (which showed 100% graft take) cannot be due to hypoperfusion but might be a result of human skin texture characteristics that present a thicker stratum corneum and greater pigmentation due to the presence of melanocytes. It suggests that laser Doppler is not suitable for comparing perfusion of skin with different textures.

From a general perspective, the animal model represents a difficult setting for the skin graft procedure. Indeed, recipient site immobilization is not possible, enhancing shear forces, while aseptic conditions under the dressings are hard to maintain. Furthermore, the inflammation phenomenon is underestimated in the immunosuppressed mice model we selected. This could have influenced the results of the TEP-CT FDG we described. The choice to perform the grafting procedure just after the acute full-thickness excision is also a matter of discussion. Although this model is classically used to assess the skin graft procedure (Michael et al., 2013; Cubo et al., 2016; Kim et al., 2018; Albanna et al., 2019; Baltazar et al., 2020; Jorgensen et al., 2020), this corresponds to a worst-case scenario compared to clinical applications where grafts are applied after granulation tissue formation, which is rich in neovessels and promotes graft take. In clinical settings, granulation tissue appears after 21 days from the hypodermis layer, which suggests that Poieskin^®^ manufacturing will need to be slightly shortened due to process duration optimization.

Different authors have already studied the *in vivo* efficacy of a bioprinting DES, but none of them were able to provide a GMP-compatible and large-size DES (Michael et al., 2013; Cubo et al., 2016; Baltazar et al., 2020; Jorgensen et al., 2020; Yang et al., 2022). Indeed, the largest reported production ever does not exceed 10 cm × 2 cm (Cubo et al., 2016), although Poieskin^®^ is an 8 cm × 5 cm sheet comparable to the handmade manufacturing method that has already been tested in humans (Blok et al., 2013; Boa et al., 2013; Blok, 2016; Germain et al., 2018; Meuli et al., 2019). Their findings are mainly focused on the histology and immunohistochemistry qualitative evaluation of the DES, with a follow-up varying from 11 days to 8 weeks. Only Jorgensen et al. (2020) reported wound closure using the epithelialization rate of 50.4% at 21 days, which appears to be lower than that of Poieskin^®^. It is important to note that none of these studies compared bioprinted DES to STSG engraftment, which is considered the reference treatment from a clinical perspective.

It is important to note that Poieskin^®^ has undergone rigorous GMP readiness assessments, including in-depth selection and risk analysis of starting and raw materials, accurate process step and cell characterization with quality controls during batch development, and a validated quality control strategy with appropriate in-process controls, all of which have been submitted and discussed with the French National Agency for Medicines and Health Products Safety (data not shown). The manufacturing process for Poieskin^®^ has been designed to meet GMP requirements for automated manufacturing equipment for ATMPs, and the automated bioprinting equipment NGB has been qualified to operate in a grade A environment. Additionally, sterilization of all specific consumables has been validated, and the entire process and manufacturing conditions of Poieskin^®^ have been validated with a dedicated aseptic process simulation test.

We believe that these results are important for the scientific community for two main reasons. First, there is a significant clinical need for large-scale DES products that have not been commercialized yet. Second, while bioprinting approaches have shown potential in recent years, most preclinical studies have been conducted on limited-size DESs that do not comply with GMP requirements. In this context, our original results support the use of Poieskin^®^ in humans as it represents a reliable and homogeneous large-size DES with a reproducible architecture and comparable features to HSTSG in terms of engraftment, safety, and surgical use.

## 5 Conclusion

In conclusion, for the first time, we described a GMP-compatible manufacturing method that produces a 3D bioprinted DES. Poieskin^®^ has consistent bioengineering and manufacturing characteristics and can be used as an alternative to STSG in clinical applications to treat full-thickness cutaneous defects.

## Data Availability

The original contributions presented in the study are included in the article/Supplementary Material further inquiries can be directed to the corresponding author.

## Abbreviations

ANOVA, analysis of variance; ALI, air–liquid interface; APAFIS, Autorisation de Projet Utilisant des Animaux à des Fins Scientifiques (Project Authorization Using Animals for Scientific Purposes); ATMP, advanced therapeutic medicinal products; BMI, body mass index; CI, confidence interval; CT, computed tomography; DES, dermo-epidermal substitute; FDG, fluorodeoxyglucose; GMP, good manufacturing practice; HSTSG: human STSG; LAB, laser-assisted bioprinting; LASER, light amplification by stimulated emission; NGB, Next-Generation Bioprinter; PET, positron emission tomography; ROI, region of interest; SD, standard deviation; STSG, split-thickness skin graft.
