# Supplementary material for: In vivo efficacy proof of concept of a large-size bioprinted dermo-epidermal substitute for permanent wound coverage
Source: Front Bioeng Biotechnol. 2023 Jul 25;11:1217655. doi: 10.3389/fbioe.2023.1217655 (PMC10407941; doi:10.3389/fbioe.2023.1217655)

**SUPPLEMENTAL TABLE 1**: Histological analysis scoring grid. Samples were scored based on the following parameters: fibroblasts morphology in the dermis, fibroblasts morphology at dermal-epidermal junction, thickness of epidermal layer, quality of dermal-epidermal junction, keratinocytes morphology and differentiation at stratum basale, at stratum spinosum, at stratum granulosum, quality of stratum corneum and general aspect of the sample. A DES was considered compliant if its total score is greater than or equal to 50%.


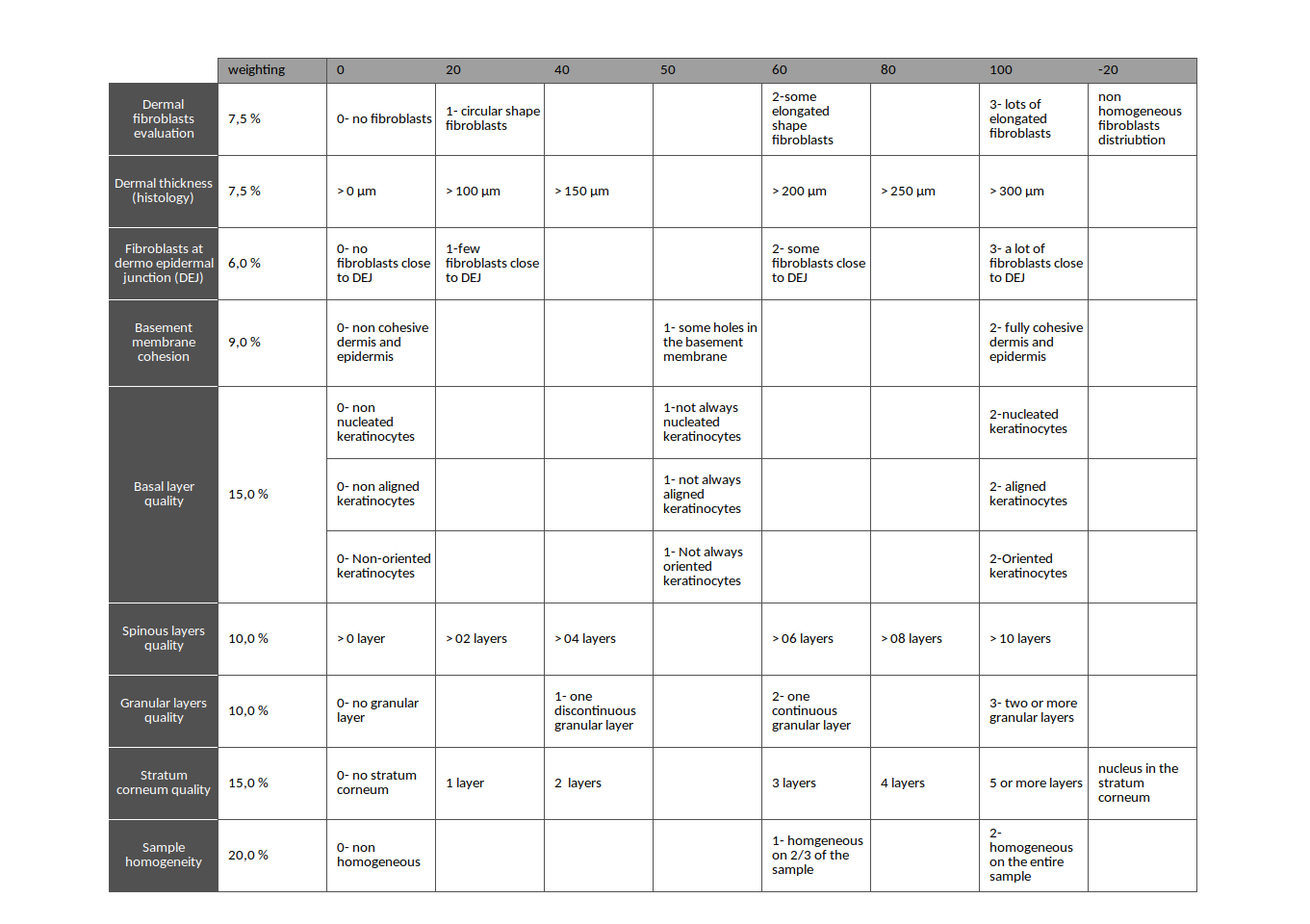

Supplement: Supplementary file 2 [file Table1.docx]
