# Supplementary material for: In vivo efficacy proof of concept of a large-size bioprinted dermo-epidermal substitute for permanent wound coverage
Source: Front Bioeng Biotechnol. 2023 Jul 25;11:1217655. doi: 10.3389/fbioe.2023.1217655 (PMC10407941; doi:10.3389/fbioe.2023.1217655)
Supplement: Supplementary file 3 [file Table2.docx]

**SUPPLEMENTAL TABLE 2A:** Biological characterization of Keratinocytes and Fibroblasts (before bioprinting step)

**SUPPLEMENTAL TABLE 2B:** Histological characteristics of both Poieskin® batches (before grafting)

| SUPPLEMENTAL TABLE 2 A |  | Batch 1 | Batch 2 |
| --- | --- | --- | --- |
| Fibroblasts | Viability (%) | 95 | 98 |
|  | Doubling population (days) | 2,19 | 2,14 |
|  | Viable nucleated cells before bio-ink formulation | compliant | compliant |
|  | Viable nucleated cells in  collagen bio-ink | compliant | compliant |
| Keratinocytes | Viability (%) | 97 | 94 |
|  | Doubling population (days) | 1,70 | 1,62 |
|  | Viable nucleated cells before bio-ink formulation | compliant | compliant |
|  | Viable nucleated cells in  collagen bio-ink | compliant | compliant |

| SUPPLEMENTAL TABLE 2 B | Batch 1 (n=5) | Batch 2 (n=3) |
| --- | --- | --- |
| Thickness (histology) | 292± 26 µm | 226 ± 31 µm |
| Scoring (%) | 75 ± 8 | 73 ± 12 |
| Detailed scoring | | |
| Fibroblasts in the dermis | 60 | 60 |
| Dermal Thickness | 80 | 67 |
| Fibroblasts at dermo-epidermal junction | 0 | 17 |
| Basale membrane cohesion | 100 | 100 |
| Epidermal Basal layer quality | 48 | 40 |
| Epidermal Spinous layer quality | 32 | 47 |
| Epidermal Granular layer quality | 80 | 67 |
| Stratum Corneum lquality | 100 | 100 |
| Sample Homogeneity | 100 | 100 |
