# Supplementary material for: In vivo efficacy proof of concept of a large-size bioprinted dermo-epidermal substitute for permanent wound coverage
Source: Front Bioeng Biotechnol. 2023 Jul 25;11:1217655. doi: 10.3389/fbioe.2023.1217655 (PMC10407941; doi:10.3389/fbioe.2023.1217655)

SUPPLEMENTAL FIGURE 1: Histological images of each non grafted Poieskin® control and one HSTSG control.

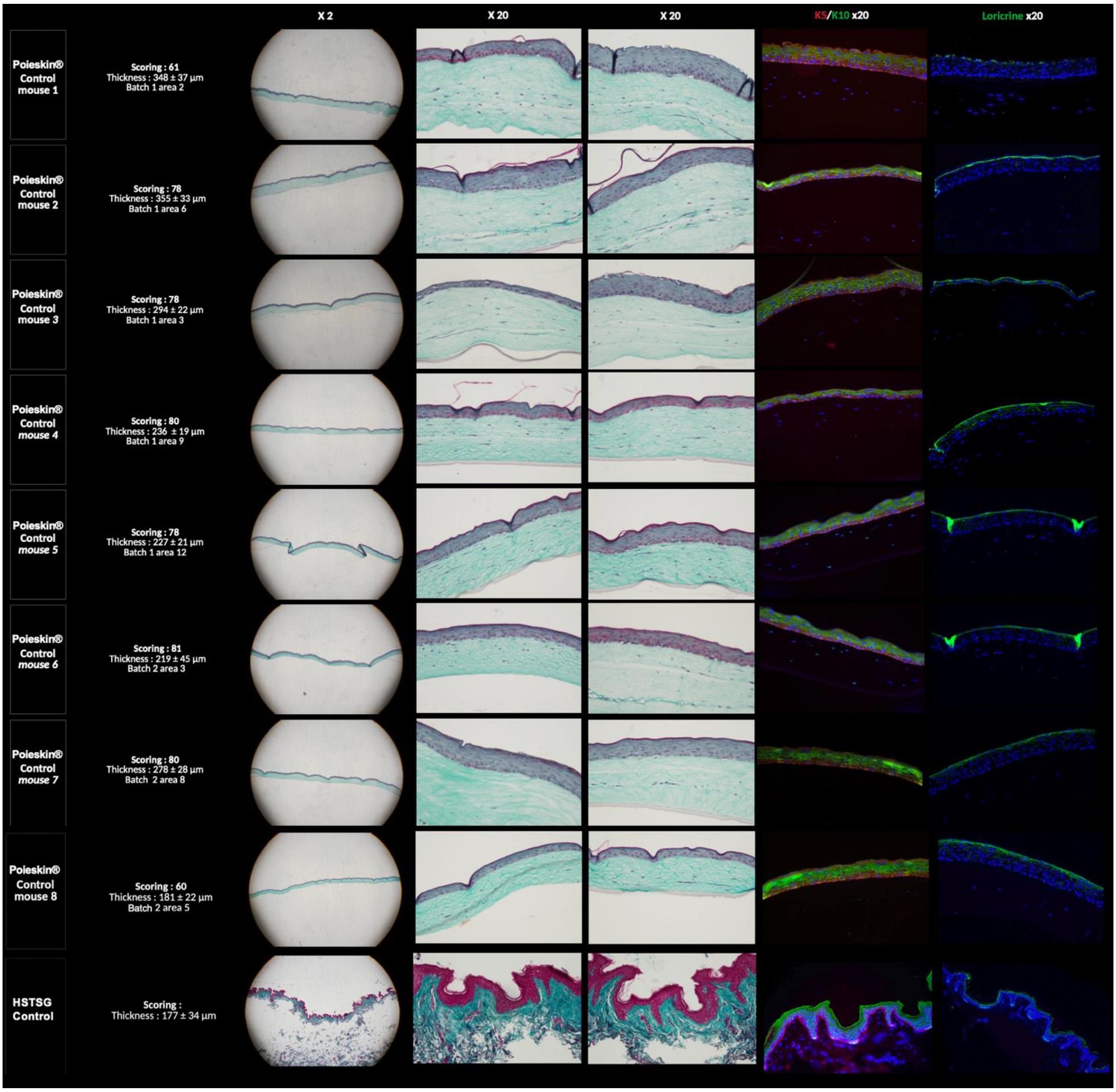

Supplement: Supplementary file 5 [file Image1.pdf]
